# Preoperative Risk Stratification in Esophageal Cancer Surgery: Comparing Risk Models with the Clinical Judgment of the Surgeon

**DOI:** 10.1245/s10434-023-13473-9

**Published:** 2023-04-29

**Authors:** Eliza R. C. Hagens, Nanke Cui, Susan van Dieren, Wietse J. Eshuis, Wytze Laméris, Mark I. van Berge Henegouwen, Suzanne S. Gisbertz

**Affiliations:** Department of Surgery, Amsterdam University Medical Centers, University of Amsterdam, Cancer Center, Amsterdam, The Netherlands

## Abstract

**Background:**

Numerous prediction models estimating the risk of complications after esophagectomy exist but are rarely used in practice. The aim of this study was to compare the clinical judgment of surgeons using these prediction models.

**Methods:**

Patients with resectable esophageal cancer who underwent an esophagectomy were included in this prospective study. Prediction models for postoperative complications after esophagectomy were selected by a systematic literature search. Clinical judgment was given by three surgeons, indicating their estimated risk for postoperative complications in percentage categories. The best performing prediction model was compared with the judgment of the surgeons, using the net reclassification improvement (NRI), category-free NRI (cfNRI), and integrated discrimination improvement (IDI) indexes.

**Results:**

Overall, 159 patients were included between March 2019 and July 2021, of whom 88 patients (55%) developed a complication. The best performing prediction model showed an area under the receiver operating characteristic curve (AUC) of 0.56. The three surgeons had an AUC of 0.53, 0.55, and 0.59, respectively, and all surgeons showed negative percentages of cfNRI_events_ and IDI_events_, and positive percentages of cfNRI_nonevents_ and IDI_events_. This indicates that in the group of patients with postoperative complications, the prediction model performed better, whereas in the group of patients without postoperative complications, the surgeons performed better. NRI_overall_ was 18% for one surgeon, while the remainder of the NRI_overall_, cfNRI_overall_ and IDI_overall_ scores showed small differences between surgeons and the prediction models.

**Conclusion:**

Prediction models tend to overestimate the risk of any complication, whereas surgeons tend to underestimate this risk. Overall, surgeons’ estimations differ between surgeons and vary between similar to slightly better than the prediction models.

**Supplementary Information:**

The online version contains supplementary material available at 10.1245/s10434-023-13473-9.

Esophageal cancer is the eighth most common cancer in the world and the sixth leading cause of death from cancer.^1^ In recent years, the introduction of neoadjuvant therapy has contributed to a better survival, and minimally invasive esophageal surgery has led to lower postoperative morbidity.^2–4^ Although postoperative mortality has decreased in the last 30 years, esophageal surgery remains a highly invasive procedure, with reported complication rates of up to 74%.^5–7^ Postoperative complications are associated with postoperative mortality, length of hospital stay, readmission rate, early cancer recurrence, long-term survival, and health-related quality of life.^8–12^ A clear understanding of the relationship between various risk factors and postoperative complications would enhance selection, counseling, and, if possible, preoperatively improve patients’ status.

Thus far, numerous prediction models have been proposed to estimate the risk of specific complications after esophagectomy;^13–23^ however, these models are not commonly used in practice and surgeons generally rely on their own clinical judgment. Hence, it remains unclear whether these existing prediction models have a higher predictive power in estimating postoperative outcome than surgeons’ judgment.

The primary aim of this study was to assess whether the available prediction models are superior to the clinical judgment of the surgeon with regard to predicting the risk of any postoperative complication, while the secondary outcome was to assess how well surgeons can predict major (Clavien–Dindo grade IIIA or higher) postoperative complications.

## Methods

### Study Design

A prospective, single center, observational cohort study was conducted at a tertiary referral hospital (Amsterdam UMC). Ethical approval was waived by the Ethical Committee. Written informed consent was obtained from all patients in this study for use of their patient data. The TRIPOD and STROBE guidelines were consulted to ensure the correct reporting of the results.^24,25^

### Study Population

Eligible patients were aged 18 years or older with resectable esophageal or gastroesophageal junction carcinoma (cT0-4aN0-3M0), and scheduled to undergo a minimally invasive transthoracic esophageal resection by one of the three surgeons. Patients were excluded in cases of a salvage esophagectomy, an esophagectomy for recurrent disease, or if nonresectable disease was found during esophagectomy.

### Treatment of Patients

All patients were treated according to the Dutch guideline.^26^ Patients were generally treated with neoadjuvant chemoradiotherapy followed by a minimally invasive transthoracic esophagectomy with a two-field lymphadenectomy. A gastric conduit reconstruction was performed with a cervical or intrathoracic anastomosis, depending on tumor characteristics and the extent of the radiotherapy field.

### Selection of Prediction Models from the Literature

A systematic search of the available literature in the PubMed and Embase databases was performed in order to identify relevant studies describing prediction models for postoperative complications after esophageal surgery; the search strategy is described in electronic supplementary Table SDC1. Studies were eligible if they described the establishment of a prediction model that predicts the occurrence of postoperative complications after an esophageal resection with gastric conduit reconstruction; however, studies were excluded if they only described a prediction model that predicts specific (i.e. only pulmonary complications) complications or only mortality after esophageal surgery.

**Fig. 1:**
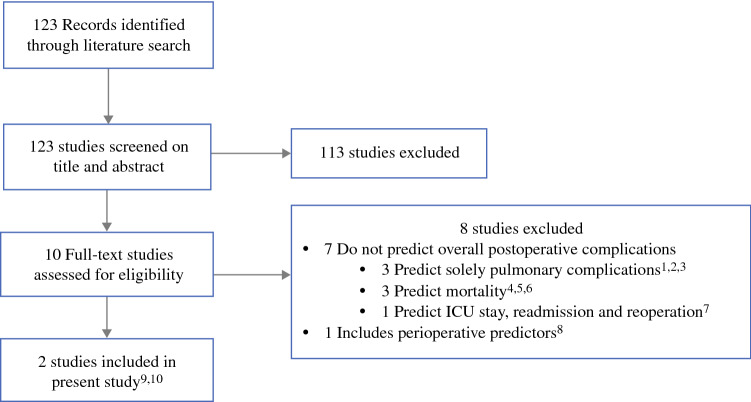
Search to identify prediction models.^10,13–19,21,23^ *ICU* intensive care unit

The literature search resulted in 123 studies. Two prediction models, one by Reeh et al. (the Preoperative Esophagectomy Risk [PER] score) and one by Lagarde et al., were identified as predictors of the risk of postoperative complications after esophagectomy.^14,27^ A flow chart of the literature search is shown in Fig. 1. A description of the included prediction models and performance of the prediction models is shown in electronic supplementary Table SDC3.

### Clinical Judgment of the Surgeon

Three surgeons (IvBH, SSG, and WJE) were asked to estimate the probability of a consecutive series of patients to develop a postoperative complication (any complication and a major complication, i.e., Clavien–Dindo grade IIIA or higher). Surgeon 1 had 14 years of experience, whereas surgeons 2 and 3 had 9 and 2 years of experience, respectively. All surgeons were blinded from the other surgeons’ response and outcome of the prediction models. One day prior to surgery, surgeons completed the Preoperative Risk Score Form (electronic supplementary Table SDC4) after studying the patient file and clinically evaluating the patient, regardless if they were operating themselves or not. The surgeons were not blinded from who was the operating surgeon. On this form, the surgeons could indicate their estimation on a 10-point scale with percentage categories for the patients to develop any or a major complication (Clavien–Dindo grade IIIA).

### Study Outcomes

The primary aim of this study was to investigate the discriminative ability of the existing prediction models compared with the accuracy of the clinical judgment of the surgeon with regard to predicting the risk of any postoperative complication, while secondary outcomes were the performance of the selected prediction models in this cohort, the performance of the surgeons, and to describe how well surgeons can predict major (Clavien–Dindo grade IIIA or higher) postoperative complications. Complications were identified and collected until 30 days post-surgery and graded according to the classification by the Esophageal Complications Consensus Group (ECCG) and the Clavien–Dindo classification.^28,29^

### Reclassification Measures

Reclassification measures were used to describe the difference between the ability of the surgeon and prediction models to predict postoperative complications.

Comparing the area under the receiver operating characteristic curves (AUCs) is the most common strategy to compare prediction models;^30^ however, comparing AUCs has proven to be insensitive to important changes in absolute risk.^31^ Therefore, reclassification measures are recommended. Using these models, patients are stratified into clinical categories based on risk in the first (reference) model, then the ability of the second (the surgeon) to more accurately reclassify individuals into higher or lower risk strata is quantified.^32,33^ Three reclassification measures were used in the current study: the net reclassification improvement (NRI), category-free NRI (cfNRI), and integrated discrimination improvement (IDI) indexes.

The NRI attempts to quantify how well a second model (in this case the surgeon) reclassifies subjects to a more appropriate risk category. The overall NRI is the sum of NRI_events_ and NRI_nonevents_. NRI ranges from −1 to 1, where 0 indicates no difference. An NRI closer to 1 correlates with a better prediction of the second model (the surgeons) and an NRI closer to −1 correlates with a better prediction by the first model. The NRI requires a threshold in risk score in order to be able to categorize patients. In this study, a threshold of 60% was chosen since the incidence of complications after esophagectomy is reported to be around 60% in Dutch centers.^34,35^ A probability of over 60% represents an increased risk. The cfNRI counts the direction of change for every individual instead of the crossing from a higher-risk group to a lower-risk group and vice versa. cfNRI values above 60% should be interpreted as a strong improvement in comparison with the reference model; those around 40% should be considered intermediate improvement and those below 20% should be considered weak improvement.^36^ Finally, IDI counts the actual change in calculated risk for each subject instead of only the direction of change, as the cfNRI does. A higher IDI correlates with better estimation by the surgeon and a negative IDI indicates a better prediction by the prediction model.

### Sample Size Calculation

To calculate the number of patients necessary for this study, the number of patients needed to develop a prediction model was used. This calculation was based on the number of degrees of freedom in the largest prediction model is this study, i.e. the study by Lagarde et al. This model includes six variables (either dichotomous or continuous). It is desirable to include a representative sample with at least 10 events and 10 nonevents per variable. Since more than half of the patients usually develop a complication, at least 60 patients without a complication were needed.^37^ Internationally, the incidence of patients with one or more postoperative complications after an esophageal resection for surgery is 60%.^34^ Therefore, the total sample size was set at 150 patients.

### Statistical Analysis

All risk factors included in the prediction models are displayed in the baseline table. To compare categorical data, the Pearson Chi-square test or Fishers exact test were used, as appropriate. The independent samples t-test was used for continuous data with normal distribution. The Mann–Whitney U test was used to compare continuous data with non-normal distribution.

The performance of the prediction models was estimated in the current dataset. For each surgeon and prediction model, the calibration (the ability to quantify the observed absolute risk) and discrimination (ability to discriminate between patients with and without an event) were described. Discrimination was examined with the AUC, and calibration was examined with the observed/expected ratio and calibration intercept and slope.

Based on calibration and discrimination, the best performing model was chosen and then compared with the clinical judgment of the surgeons using reclassification measures (NRI, cfNRI, and IDI) in addition to quantifying the difference between AUCs. Missing data were handled with single imputation. All *p*-values were based on a two-sided test and a *p*-value <0.050 was considered statistically significant. Data were analyzed using SPSS for windows, version 25 (IBM Corporation, Armonk, NY, USA) and R version 3.3.3 (R Foundation for Statistical Computing, Vienna, Austria).

## Results

A total of 208 patients underwent esophagectomy between March 2019 and July 2021. A total of 49 patients were excluded: 18 patients because none of the surgeons had estimated the postoperative complication risk, 17 patients underwent salvage esophagectomy, 5 were intraoperatively found to have nonresectable disease, and 9 underwent an open esophagectomy. Therefore, in total 159 patients were included in the present study. Overall, 88 of 159 patients (55%) developed postoperative complications within the first 30 days. Of those, 48 patients (55%) developed a minor complication (Clavien–Dindo lower than grade III), whereas 40 patients (45%) developed a major complication (Clavien–Dindo grade IIIA or higher). Clinicopathological characteristics were comparable between patients with and without complications (Table 1). Table 2 shows the incidence of specific postoperative complications and severity.

**Table 1:** Clinicopathological characteristics of all patients included in this study

	All patients	Complication	No complication	*p*-Value
	[*n* = 159]	[*n* = 88]	[*n* = 71]	
Age, years, mean (SD)^b^	66.4 ± 10.7	66.7 ± 8.6	66.0 ± 12.5	0.151
Female	27 (17.0)	14 (15.9)	13 (18.3)	0.689
*Ethnicity*				0.194
White	143 (89.9)	75 (85.2)	68 (95.8)	
Turkish	4 (2.5)	1 (1.4)	3 (4.3)	
Asian	3 (1.9)	2 (2.3)	1 (1.4)	
Black	2 (1.3)	2 (2.3)	0	
Others/mixed	4 (2.5)	3 (3.4)	1 (1.4)	
*BMI*				0.375
Underweight (<18.5 kg/m^2^)	1 (0.6)	1 (11.4)	0	
Normal weight (18.5–24.9 kg/m^2^)	67 (42.1)	33 (37.5)	34 (47.9)	
Overweight (25–29.9 kg/m^2^)	67 (42.1)	38 (43.2)	29 (40.8)	
Obesity (≥30 kg/m^2^)	24 (16.0)	16 (18.2)	8 (11.3)	
*Comorbidities*				
History of CVA/TIA^b^	5 (3.1)	4 (4.5)	1 (1.4)	0.382
History of MI^b^	14 (8.8)	7 (8.0)	7 (9.9)	0.674
COPD	10 (6.3)	9 (10.3)	1 (1.4)	0.023
Diabetes mellitus	24 (16.0)	14 (15.9)	10 (14.1)	0.922
Q-waves and/or ST-T changes on ECG^b^	18 (11.3)	11 (12.5)	7 (9.9)	0.601
RCRI score, mean (SD)^a^	1.4 (0.7)	1.7 (0.7)	1.4 (0.6)	0.103
PER score, mean (SD)^a^	3.5 (0.7)	3.5 (0.8)	3.4 (0.6)	0.037
*Preoperative lung function*^a^				
FEV_1_, L/s, mean (SD)^b^	3.4 (1.2)	3.3 (1.2)	3.5 (1.3)	0.274
FEV_1_, mean (SD)^b^	100.8 (18.1)	98.7 (18.3)	103.4 (17.8)	0.098
VC, mean (SD)^b^	109.7 (17.0)	107.7 (17.4)	112.3 (16.3)	0.090
MELD score, mean (SD)^a^	6.9 (1.5)	7.2 (1.6)	6.4 (1.1)	0.717
*ASA*				0.162
I	17 (10.7)	6 (6.8)	11 (15.5)	
II	96 (60.4)	52 (59.1)	44 (62.0)	
III	45 (28.3)	29 (33.0)	16 (22.5)	
V	1 (0.6)	1 (1.1)	0	
*Clinical T stage*				0.107
cT0	4 (2.5)	1 (1.1)	3 (4.2)	
cT1	9 (5.7)	5 (5.7)	4 (5.6)	
cT2	24 (15.1)	15 (17.0)	9 (12.7)	
cT3	122 (76.7)	67 (76.1)	55 (77.5)	
*Clinical N stage*				0.626
cN0	65 (40.9)	34 (38.6)	31 (43.7)	
cN1	61 (38.4)	33 (37.5)	28 (39.4)	
cN2	32 (20.1)	20 (22.7)	12 (16.9)	
cN3	1 (0.6)	1 (1.1)	0	
*Neoadjuvant treatment*				0.949
None	7 (4.4)	4 (4.5)	3 (4.2)	
Chemotherapy	8 (5.0)	4 (4.5)	4 (5.6)	
Chemoradiation	144 (90.6)	80 (90.9)	64 (90.1)	
*TRG*				0.644
1	42 (26.4)	20 (22.7)	22 (31.0)	
2	43 (27.0)	23 (26.1)	20 (28.2)	
3	40 (25.2)	26 (29.5)	14 (19.7)	
4	21 (13.2)	12 (13.6)	9 (12.7)	
5	6 (3.8)	4 (4.5)	2 (2.8)	
Intrathoracic anastomosis	140 (88.1)	74 (84.1)	66 (93.0)	0.146
*Histology*				0.594
Squamous cell carcinoma	24 (15.1)	15 (17.0)	9 (12.7)	
Adenocarcinoma	127 (79.9)	68 (77.2)	59 (83.1)	
Other	8 (5.0)	5 (5.7)	3 (4.2)	

Data are expressed as *n* (%) unless otherwise specified

*BMI* body mass index, *ASA* American Society of Anesthesiologists, *TRG* tumor regression grade, *COPD* chronic obstructive pulmonary disease, *MI* myocardial infarction, *CVA* cerebrovascular accident, *TIA* transient ischemic attack, *FEV*_*1*_ forced expiratory volume in 1 s, *MELD* model of end-stage liver disease, *RCRI* revised Cardiac Risk Index, *ECG* electrocardiogram, *PER* Preoperative Esophagectomy Risk

^a^Predictor in the model by Reeh et al.

^b^Predictor in the model by Lagarde et al.

**Table 2:** Incidence of postoperative complications and severity [*n* = 159]

	*N* (%)
Any complication	88 (55.3)
More than one complication	49 (30.8)
*Type of complication*	
Conduit necrosis	1 (0.6)
Type I	1 (0.6)
Neurological complications	11 (6.9)
Pulmonary complications	51 (32.1)
Cardiac complications	33 (20.8)
Anastomotic leakage	21 (13.2)
Type I	4 (2.5)
Type II	12 (7.5)
Type III	5 (3.1)
Chyle leakage	20 (12.6)
Type I	18 (11.3)
Type II	1 (0.6)
Type III	1 (0.6)
Renal complications	3 (1.9)
Sepsis	7 (4.4)
Delayed gastric emptying	4 (2.5)
*Clavien–Dindo grade*	
0	71 (45.3)
I	9 (5.6)
II	39 (24.5)
IIIa	10 (6.3)
IIIb	6 (3.8)
IVa	14 (8.8)
IVb	7 (4.4)
V (death)	3 (1.9)

Complications were graded according to the classification by the esophageal complications consensus group (ECCG) and the Clavien–Dindo classification^28,29^

### Performance of Prediction Models

Of the 106 patients with a PER score classified as ‘low risk’ by Reeh et al., 56 (52%) developed a complication; of the 35 patients with a PER score of ‘medium risk’, 20 (57%) developed a complication; and of the 18 patients with a PER score of ‘high risk’, 12 patients (67%) developed a complication. Median PER scores for patients with and without complications are shown in Table 3.

**Table 3:** Median risk scores by prediction models and surgeons

	Patients with complications	Patients without complications
*Median model score (IQR)*		
Prediction models		
Model by Reeh et al.	4 (3–4)	3 (3–4)
Model by Lagarde et al.	23 (20–26)	22 (19–25)
Expected probability^a^	82% (79–87%)	78% (76–86%)
*Median risk score (IQR)*		
Estimation by surgeon		
Surgeon 1	55% (IQR 25–65%)	45% (IQR 5–55%)
Surgeon 2	55% (IQR 35–75%)	55% (IQR 35–75%)
Surgeon 3	65% (IQR 45–65%)	55% (IQR 35–65%)

Prediction model by the Reeh et al. score ranges from 2 to 8; prediction model by the Lagarde et al. score ranges from 0 to 42

^a^Expected probability corresponding to the model score by Lagarde et al.

*IQR* interquartile range

Using the model by Lagarde and colleagues, patients who did not develop complications had a median score of 22 (interquartile range [IQR] 19–25), and those who did develop complications had a median score of 23 (IQR 20–26) (Table 3). The performance of both models to predict any complication are displayed in Table 4. The prediction model by Lagarde et al. had better performance and was therefore compared with the risk estimation by the surgeons.

**Table 4:** Performance of prediction models and surgeons in predicting any complication

	Area under the curve (95% CI)	Observed/expected ratio	Calibration intercept	Calibration slope
*Prediction models*				
Model by Reeh et al.	0.54 (0.45–0.63)	NA	NA	NA
Model by Lagarde et al.	0.56 (0.47–0.65)	0.69	−0.39	0.40
*Estimation by the surgeon*				
Surgeon 1	0.53 (0.45–0.62)	1.34	0.37	0.23
Surgeon 2	0.55 (0.46–0.64)	1.04	0.21	0.15
Surgeon 3	0.59 (0.50–0.68)	1.02	0.17	0.25

*NA* not applicable for this model, *CI* confidence interval

### Risk Estimation by the Surgeons

Risk estimations were made by three surgeons using the preoperative form for risk assessment. For surgeon 1, patients with a complication had a median risk score of 55% (IQR 25–65%) and patients without a complication had a median risk score of 45% (IQR 5–55%); for surgeon 2, patients with and without complications had a median risk score of 55% (IQR 35–75%) and 55% (IQR 35–75%), respectively; and for surgeon 3, patients with and without complications had a median risk score of 65% (IQR 45–65%) and 55% (IQR 35–65), respectively (Table 3). The discriminative ability and calibration measures for each surgeon are displayed in Table 4.

### Comparison of the Prediction Model and Risk Estimation by the Surgeon in Predicting Any Complication

Surgeons 1 and 2 had a lower AUC and surgeon 3 had a higher AUC than the prediction model by Lagarde et al., although these differences were small and were not statistically significant (Table 5). The observed/expected ratio was 0.69 for the model by Lagarde et al., indicating an overestimation of the risk of complications, whereas the observed/expected ratios for the surgeons where all >1, indicating an underestimation of the risk of complications (Table 4).

**Table 5:** Comparison of the prediction model and clinical judgment by surgeons

	Surgeon 1	Surgeon 2	Surgeon 3
NRI, % (95% CI)			
NRI_events_	−97.73 (−100.87 to −94.59)	−51.14 (−61.62 to −40.65)	−44.32 (−54.90 to −33.74)
NRI_nonevents_	90.14 (80.55–99.74)	52.11 (38.69–65.54)	61.97 (49.27–74.68)
NRI	−7.59 (−17.24 to 2.07)	0.98 (−13.61 to 15.56)	17.65 (1.21–34.10)
cfNRI, % (95% CI)			
cfNRI_events_	−100.00 (−100.00 to −100.00)	−75.00 (−87.00 to −63.00)	−77.27 (−89.03 to −65.52)
cfNRI_nonevents_	94.37 (84.32–104.41)	71.83 (54.74–88.93)	77.46 (66.48–88.45)
cfNRI	−5.63 (−15.68 to 4.41)	−3.17 (−22.69 to 16.35)	0.19 (−14.83 to 15.22)
IDI, % (95% CI)			
IDI_events_	−50.26 (−56.29 to −44.23)	−27.14 (−31.78 to −22.50)	−25.77 (−30.99 to −20.56)
IDI_nonevents_	50.26 (43.87–56.66)	27.73 (21.28–34.18)	26.60 (20.93–32.28)
IDI	−3.01 (−10.23 to 4.21)	0.59 (−6.50 to 7.68)	0.83 (−6.42 to 8.07)
Area under the curve			
AUC_model_	0.56 (0.46–0.67)	0.56 (0.46–0.67)	0.56 (0.46–0.67)
AUC_surgeon_	0.53 (0.45–0.62)	0.55 (0.48–0.63)	0.59 (0.50–0.68)
AUC difference (*p*-value)	0.658	0.841	0.626

*NRI* net reclassification improvement (NRI_events_ + NRI_nonevents_), *cfNRI* category-free NRI (cfNRI_events_ + cfNRI_events_), *IDI* integrated discrimination improvement (IDI_events_ + IDI_nonevents_), *AUC* area under the receiver operating characteristic curve, *CI* confidence interval

This was reflected in negative NRI_events_, cfNRI_events_ and IDI_events_ scores and positive NRI_nonevents_, cfNRI_nonevents_ and IDI_nonevents_ scores (Table 5).

Overall, the NRI for surgeons 1, 2, and 3 were −8%, 1%, and 18%, respectively, indicating improvement for surgeon 3 compared with the prediction model, and a similar estimation compared with the model for surgeons 1 and 2. The cfNRI showed no improvement in the estimation from all surgeons (−6%, −3%, and 0% for surgeons 1, 2, and 3, respectively). The IDI showed small differences between the surgeons and the prediction model (−3%, 1%, and 1%, respectively). All reclassification measures are detailed in Table 5.

### Risk Estimation by the Surgeon in Predicting Major (Clavien–Dindo Grade IIIA or Higher) Complications

The median estimated risk for patients with a major postoperative complication by surgeons 1, 2, and 3 was 35% (IQR 15–35%), 15% (5–35%), and 45% (35–55%), respectively, and 25% (15–35%), 15% (5–35%), and 35% (18–55%) for patients without major postoperative complications. Discrimination and calibration are shown in Table 6.

**Table 6:** Performance of surgeons in predicting major complications

	Area under the curve (95% CI)	Observed/expected ratio	Calibration intercept	Calibration slope
*Estimation by surgeon*				
Surgeon 1	0.59 (0.48–0.70)	0.86	−0.66	0.50
Surgeon 2	0.55 (0.45–0.66)	1.17	−0.79	0.22
Surgeon 3	0.59 (0.49–0.69)	0.65	−0.89	0.44

*CI* confidence interval

## Discussion

Risk stratification has been a hot topic in esophageal cancer surgery for many years. We investigated whether available prediction models are superior compared with the clinical judgment of the surgeon with regard to predicting the risk of postoperative complications after esophagectomy. Surgeons 1 and 2 performed similar to the prediction models and surgeon 3 performed slightly better. Moreover, the prediction models tended to overestimate the risk of any complication, whereas all surgeons tended to underestimate the risk of any complication. When estimating the risk for major complications (Clavien–Dindo grade IIIA or higher), three surgeons had an AUC of around 0.5 and poor calibration. It is of clinical relevance to identify high-risk patients in order to improve informing about their risks for complication. High-risk patients could be monitored more thoroughly postoperatively and perhaps a lower threshold for postoperative diagnostics or treatment would be justified. To our knowledge, this is the first study evaluating prediction models in comparison with the clinical judgment of specialized surgeons with regard to predicting the risk of postoperative complications after esophagectomy.

The performance of the model by Reeh and colleagues differed considerably between the current study and the original study.^14^ The poor predictive performance could be explained by the differences in patient groups and treatment characteristics between both cohorts. Almost all patients in the current study received neoadjuvant chemotherapy or chemoradiotherapy, whereas none of the patients in the study by Reeh et al. were treated neoadjuvantly. Moreover, every patient in the current study underwent a transthoracic esophagectomy versus less than half of the patients in the development cohort. Neoadjuvant therapy and a transthoracic approach can both negatively affect postoperative outcomes.^38,39^

Our results differ from the model proposed by Lagarde et al. regarding performance measures. We found an AUC of 0.56, whereas Lagarde et al. reported an AUC of 0.65 in their test cohort and 0.64 in the external validation cohort.^40^ Patients in the study by Lagarde et al. were all operated by the open approach and were not treated with neoadjuvant therapy, whereas in the current study, all patients were treated by a minimally invasive procedure and treated neoadjuvantly. These differences between the developmental cohort and the current study cohort might explain the differences in performance.

Clinical judgment, considering comorbidities, preoperative tests, and an estimation of a patient’s ability to withstand the physical damage of surgery, are all essential in patient selection for esophagectomy. Multiple studies have demonstrated that the surgeons’ clinical assessment is a good predictor of postoperative complications in major gastrointestinal surgery and is even more accurate than the POSSUM score.^41,42^ Our results revealed heterogeneity between the surgeons’ clinical judgment. All surgeons underestimated the risk of complications, which is also seen in another study evaluating surgeons’ assessment in different types of surgery.^43^ One surgeon had an acceptable AUC of 0.59, whereas others performed poorly, with an AUC ranging from 0.53 to 0.59. The lack of agreement could be explained by different factors on which surgeons base their clinical judgment or the difference in years of experience, since the most experienced surgeon had the best AUC.

Of all patients, 25% developed a major complication. Surgeons 1 and 3 performed better than surgeon 2 in predicting major complications, with an AUC of 0.59, and higher median risk scores for patients with major complications compared with patients without. Calibration also varied widely between surgeons, yet all surgeons had poor calibration measures. No other studies have compared surgeons’ assessment with prediction models in assessing major complications after esophagectomy. D'Journo et al. developed and validated a risk prediction model of death within 90 days after esophagectomy.^44^ This model showed good discriminative ability, with an AUC of 0.64 in the validation cohort. Future studies could compare surgeons’ assessment with this model or develop a new prediction model specifically focusing on major complications.

All surgeons had negative NRI_events_, cfNRI_events_ and IDI_events_, and positive NRI_nonevents_, cfNRI_nonevents_ and NRI_nonevents_ percentages. This indicates that surgeons underestimate the risk of a complication compared with the prediction model. The overall NRI percentage was similar to 0 for surgeons 1 and 2, and significantly positive for surgeon 3, indicating that only surgeon 3 was better at predicting overall complications than the prediction model, when utilizing a threshold of 60% risk. However, when comparing the surgeons with the prediction model without a threshold (cfNRI), one surgeon showed a weak improvement and two surgeons showed a small diminishment. When quantifying the improvement of reclassification by surgeons compared with the prediction models, the IDI scores were positive for two surgeons and negative for one surgeon, but all were close to 0%. Thus, surgeons perform similar to the prediction model in predicting overall complications based on IDI and cfNRI. To date, no studies exist that evaluate the clinical judgment of surgeons in predicting postoperative morbidity after esophagectomy to compare our results with. However, a systematic review assessing the accuracy with which surgeons can predict outcomes following many different types of surgery, including gastrointestinal, found that the surgeons' prediction of general morbidity was good and was equivalent to or better than pre-existing prediction models.^45^

Evaluating the relationship between experience and accuracy in predicting complications showed that surgeon 3, the most experienced surgeon, had a higher median risk score for patients with complications, the highest AUC, and overall better calibration measures compared with surgeons 2 and 3. Surgeon 3 with the least experience has less favorable outcomes than surgeon 2. These data show that there is a trend of surgeons with more experience in predicting complications more accurately than less experienced surgeons, although no statistical tests could be performed reliably due to the low number of participants. These results are in line with another study that showed that senior surgeons were superior in predicting outcomes.^46^

The present study has some limitations. First, there were few prediction models designed specifically for predicting postoperative complications after esophagectomy. Furthermore, our single-institution study provides less generalizable results. Moreover, most of these models were constructed before the implementation of minimally invasive transthoracic surgery and/or neoadjuvant therapy, which makes these models less generalizable to current practice. This also indicates that there is a need for an up-to-date prediction model. Furthermore, surgeons were not able to be blinded as to who the operating surgeon was since this team of surgeons always operates together and the scores were completed 1 day before the actual surgery. Additionally, the 10-point scale for the surgeons to indicate their estimated risk for a patient to develop a postoperative complication is a nonvalidated tool and was chosen because it is straightforward but still enables reclassification. However, this form can feel counterintuitive to some who would prefer a dichotomous scale or a visual analogue scale. The risk of complications should be weighed against the ‘risk’ of a complete pathological response, in which case surgery could be omitted. In this study, complication and pathological complete response (pCR) rates were 25% and 26%, respectively. Unfortunately, it is still not possible to reliably predict a cPR, as 60% of patients in a recent study still had vital tumor even though the clinical response evaluation was negative.^47^ Perhaps in the future, with the evolving of (imaging) techniques, this rate will improve and patients can be safely offered active surveillance.

This study has found that generally, surgeons underestimate the risk of complications and the prediction models overestimate the risk of complications. When comparing both, two surgeons predicted complications similar to the prediction model and one surgeon predicted complications slightly better than the prediction model. The surgeon’s assessment is therefore important when counseling patients about the risks of esophageal surgery in addition to prediction models. However, there was a large heterogeneity between the risk estimations between surgeons. This implicates that both prediction models and the clinical judgment of the surgeons are equally useful, and possibly combining both might lead to the best risk assessment. Discussing patients in multidisciplinary teams with multiple surgeons and other specialists might benefit the risk estimation of the surgeon. One study evaluating the ability of the surgeon to predict complications among different types of surgery incorporated the surgeons’ assessment in a previously developed multifactorial model and found an improved discriminative ability.^43^ More so, evidence suggests that exposure to pre‐existing prediction models leads to less varied and more accurate judgments of operative risk among surgeons and thus should be used in tandem with their gut feeling.^45,48^ Therefore, further studies could validate our findings and incorporate surgeons’ assessment in prediction models, or combine prediction models specifically for esophageal surgery with the clinical judgment of the surgeon. Given the finding that surgeons generally underestimated the risk of postoperative complications, it would be valuable to assess if providing feedback to the surgeons would help improve their estimation. Another interesting endpoint could be if the clinical judgment of the surgeon directly after surgery (incorporating blood loss, quality of the gastric conduit) changes their estimation. This does not facilitate the possibility to better inform patients about the risk of complications, but has the benefit of identifying high-risk patients. In addition, since major complications result in more postoperative mortality and decreased quality of life, future studies should therefore not only focus on predicting any complication but also on predicting major complications. We aim to conduct a follow-up study developing a new prediction model that takes into account the current treatment of neoadjuvant therapy and minimally invasive surgery. We will consecutively analyze if incorporation of the estimation by the surgeon would benefit this model. One of the endpoints in this follow-up study would be the inter-surgeon variability in predictions, and also to identify factors that contribute to a correct prediction by surgeons.

## Conclusion

This study demonstrated that surgeons’ assessment differs between surgeons and varies between similar to slightly better than the prediction models in predicting the risk of postoperative complications after esophageal cancer surgery. Prediction models could be used in tandem with surgeons’ own risk estimation. Future studies are required in order to assess the benefit of incorporating the surgeon’s assessment in prediction models to reach a higher level of predicting outcomes in this patient group with high chances of postoperative complications.

## Supplementary Information

Below is the link to the electronic supplementary material.

Supplementary file1 (DOCX 14 kb)

Supplementary file2 (DOCX 16 kb)

Supplementary file3 (DOCX 16 kb)
