# Supplementary material for: Preoperative Risk Stratification in Esophageal Cancer Surgery: Comparing Risk Models with the Clinical Judgment of the Surgeon
Source: Ann Surg Oncol. 2023 Apr 29;30(8):5159–69. doi: 10.1245/s10434-023-13473-9 (PMC10319689; doi:10.1245/s10434-023-13473-9)
Supplement: Supplementary file 1 — Supplementary file1 (DOCX 14 kb) [file 10434_2023_13473_MOESM1_ESM.docx]

| **Table SDC1.** Literature search to identify prediction models |
| --- |
| Database: OVID MEDLINE 2009 to Present |
| Search date: 3 December 2018 |
| 1. "Esophagectomy"[Mesh] OR esophageal resection[tiab] OR esophagectomy*[tiab] OR esophageal surgery[tiab] OR McKeown[tiab] OR Ivor Lewis[tiab] |
| 1. (((“preoperative” [Title/abstract]) AND (“risk”[Title/abstract]) AND (“stratification”[Title/abstract])) OR ((“scoring”[Title/abstract]) AND (“system”[Title/abstract])) OR ((“risk”[Title/abstract]) AND (“score”[Title/abstract]))) |
| #1 and #2 |
