# Supplementary material for: Preoperative Risk Stratification in Esophageal Cancer Surgery: Comparing Risk Models with the Clinical Judgment of the Surgeon
Source: Ann Surg Oncol. 2023 Apr 29;30(8):5159–69. doi: 10.1245/s10434-023-13473-9 (PMC10319689; doi:10.1245/s10434-023-13473-9)
Supplement: Supplementary file 2 — Supplementary file2 (DOCX 16 kb) [file 10434_2023_13473_MOESM2_ESM.docx]

**Table SDC3.** Prediction models that predict postoperative complications after oncological esophageal surgery.

|  | Reeh, *et al* | Lagarde, *et al* |
| --- | --- | --- |
| **Year** | 2016 | 2008 |
| **Study population** | Patients with esophageal cancer treated by esophageal resection between 1994 and 2007. | Patients with esophageal cancer treated by esophageal resection between 1993 and 2005. |
| **Establishment of the model** | The PER score was based on three validated risk indices for three organ systems:  1. The Revised Cardiac Risk index (RCRI) 2. The model for end-stage liver disease (MELD) score 3. the pulmonary function test (PFT).  The assessment of the different organ systems resulted in risk  points that were summed to PER score groups. If none of the  organ systems were impaired the patient was assigned to the PER  group 1. Moderate impairment of 1 organ system resulted in  PER group 2 classification and moderate impairment of 2 or  more organ systems or severe impairment of 1 organ system  directed patients to PER group 3. | Various readily available  preoperative potential predictors were selected by a panel  of four contributing authors. All these variables which had a p value of <0.10 in univariable analyses were entered in multivariable analysis. To establish the final, model a proportional odds model (ordinal logistic regression) was made using backward elimination (<0.1 to stay in the model). |
| **Predictors included in the scoring model** | RCRI (High risk surgery, history of ischemic heart disease, history of congestive heart failure, history of cerebrovascular disease, pre-operative treatment with insulin, pre-operative creatinine); PFT (vital capacity, FEV1); MELD (Dialysis at least twice per week, Creatinine, Bilirubin, INR, Sodium) | Transthoracic esophagectomy, Q-waves and ST-T changes on ECG, history of CVA/TIA, age, preoperative FEV1, history of myocardial infarction |
| **Outcome measures of the model** | PER 1: low-risk group PER 2: intermediate-risk group  PER 3: high risk group | A continuous score which is correlated to a % probability for no, minor or major complications. |
| **Performance of the model** | 9% of the patients with PER 1 developed complications, 18% of the patients with PER 2 and 27% of the patients with PER 3. No other performance measures reported. No external validation. | Internal validation showed a C-statistic of 0.65 and the goodness of fit test for ordinal models had p value of 0.366. External validation of consecutive new series of 95 patients showed a significant difference between all three complication groups and the mean risk score (p<0.05). C statistic was 0.66 and the Hosmer Lemeshow Goodness of fit test showed a p value of 0.626. A calibration plot was also provided. |
