# Supplementary material for: Preoperative Risk Stratification in Esophageal Cancer Surgery: Comparing Risk Models with the Clinical Judgment of the Surgeon
Source: Ann Surg Oncol. 2023 Apr 29;30(8):5159–69. doi: 10.1245/s10434-023-13473-9 (PMC10319689; doi:10.1245/s10434-023-13473-9)
Supplement: Supplementary file 3 — Supplementary file3 (DOCX 16 kb) [file 10434_2023_13473_MOESM3_ESM.docx]

**Table SDC4.** Preoperative form for risk assessment.

| Patient sticker or name and MDN number: | **Surgeon:**  □ Prof. dr. M.I. van Berge Henegouwen  □ Dr. S.S. Gisbertz □ Dr. W.J. Eshuis  □ Dr. W. Laméris  □ Other (please specify):…………. |
| --- | --- |
| Date of assessment: |  |
| What is the estimated risk of… | |
| …**any** postoperative **complication** for this patient: | …a **MAJOR** postoperative c**omplication** (Clavien Dindo grade ≥IIIa): |
| □ 0-10 % □ 11-20% □ 21-30% □ 31-40% □ 41-50% □ 51-60% □ 61-70% □ 71-80% □ 81-90% □ 91-100% | □ 0-10 % □ 11-20% □ 21-30% □ 31-40% □ 41-50% □ 51-60% □ 61-70% □ 71-80% □ 81-90% □ 91-100% |

| **Clavien Dindo Classification** | |
| --- | --- |
| Grade I | Any deviation from the normal postoperative course without the need for pharmacological treatment or surgical, endoscopic and radiological interventions. This grade also includes wound infections opened at the bedside. |
| Grade II | Requiring pharmacological treatment with drugs other than such allowed for grade I complications. Blood transfusions and total parenteral nutrition are also included. |
| Grade III | Requiring surgical, endoscopic or radiological intervention |
|  | \| IIIa: \| Intervention not under general anesthesia \| \| --- \| --- \| \| IIIb: \| Intervention under general anesthesia \| |
| Grade IV | Life-threatening complication (including Central Nervous System complications)  requiring IC/ICU-management |
|  | \| IVa: \| Single organ dysfunction (including dialysis) \| \| --- \| --- \| \| IVb: \| Multi organ dysfunction \| |
